# Functional Outcomes Following Cytoreductive Surgery and Hyperthermic Intraperitoneal Chemotherapy: A Prospective Cohort Study

**DOI:** 10.1245/s10434-022-12691-x

**Published:** 2022-10-28

**Authors:** Preet G. S. Makker, Cherry E. Koh, Nabila Ansari, Nicole Gonzaga, Jenna Bartyn, Michael Solomon, Daniel Steffens

**Affiliations:** 1 Surgical Outcomes Research Centre (SOuRCe), Royal Prince Alfred Hospital, Sydney, NSW Australia; 2 Faculty of Medicine and Health, Central Clinical School, The University of Sydney, Sydney, NSW Australia; 3 Department of Colorectal Surgery, Royal Prince Alfred Hospital, Sydney, NSW Australia; 4 RPA Institute of Academic Surgery, Royal Prince Alfred Hospital, Sydney, NSW Australia

## Abstract

**Background:**

Pre-operative physical status and its association with post-operative surgical outcomes is poorly understood in patients with peritoneal malignancy who undergo cytoreductive surgery (CRS) with hyperthermic intraperitoneal chemotherapy (HIPEC). The aims of this study were to determine the pre-operative physical function in patients having CRS-HIPEC and investigate the association between physical function and post-operative outcomes.

**Patients and Methods:**

Patients undergoing CRS-HIPEC between 2017 and 2021 were recruited at a single quaternary referral hospital in Sydney, Australia. The primary physical function measures were the 6-min walk test (6MWT) and the five-times sit to stand test (5STS). Data were collected pre-operatively and at post-operative day 10, and were analysed according to pre-operative patient characteristics and post-operative outcomes such as length of hospital stay (LOS) and complications.

**Results:**

The cohort of patients that participated in functional assessments consisted of 234 patients, with a median age of 56 years. Patients having CRS-HIPEC performed worse on the 6MWT pre-operatively compared with the general Australian population (*p* < 0.001). Post-operatively, these patients experienced a further deterioration in 6MWT and 5STS performance and the degree of the post-operative decline in function was associated with post-operative morbidity. A higher level of pre-operative physical function was associated with shorter LOS and minor post-operative complications.

**Conclusions:**

Patients who have undergone CRS-HIPEC were functionally impaired pre-operatively compared with the general population and experience a further deterioration of physical function post-operatively. A higher level of pre-operative physical function is associated with minor post-operative morbidity, which is highly relevant for pre-operative optimisation of patients with cancer.

**Supplementary Information:**

The online version contains supplementary material available at 10.1245/s10434-022-12691-x.

The management of peritoneal malignancy remains a challenging area in surgical oncology. The recent emergence of cytoreductive surgery (CRS) and hyperthermic intraperitoneal chemotherapy (HIPEC) as a treatment strategy over systemic chemotherapy or palliative care for selected patients has led to superior 5-year survival rates, better disease control and improvement in longer term health-related quality of life (QoL).^1–4^ CRS is performed on primary or metastatic peritoneal disease arising from cancers of the appendix, colon, rectum, mesothelium and ovary, with the goal of removing all visible disease using a combination of visceral resections and peritonectomy procedures.^5^ HIPEC involves direct administration of a heated chemotherapy agent intraoperatively following cytoreduction to treat any microscopic disease.^6^ It is estimated that between 29,000 and 41,000 CRS-HIPEC procedures are performed in the USA every year.^7^

Whilst patient survival rates from peritoneal malignancy have improved with a CRS-HIPEC approach for selected patients, there continues to be significant morbidity associated with this procedure.^1,8^ Therefore, there is growing interest in improving outcomes in this patient population.^9^ Post-operative complications, length of hospital stay and 30-day mortality are either comparable or favourable in patients having CRS-HIPEC when compared with other high-risk oncological procedures such as hepatectomy, pancreaticoduodenectomy and oesophagectomy.^8^ In addition, several studies assessing short- and long-term QoL in CRS-HIPEC populations have found a significant short-term decline in QoL, with a return to baseline levels ranging from 6 months to 2 years after surgery.^10–13^ Studies investigating level of physical function through patient-reported measures have reported a short-term post-operative decline in function.^10,11,13,14^ Patient-reported outcomes are prone to bias and variability between testing centres, leading to inconclusive results and a lack of generalisability.^15^ Studies measuring physical function using objective measures are lacking in patients having CRS-HIPEC.

The 6-min walk test (6MWT) and five-times sit to stand test (5STS) are clinically validated tools for measuring functional capacity and lower limb function, which are relevant dimensions for determining pre-operative fitness for surgery and post-operative recovery. There are limited studies that report objectively measured functional outcomes in patients with abdominal and pelvic cancers using 6MWT and 5STS,^16–19^ and to the authors’ knowledge, no studies to date utilise these tools in the context of peritoneal malignancy and CRS-HIPEC. A previously published cohort study investigated the clinical application of 6MWT and 5STS in the pre-operative and post-operative setting in patients undergoing pelvic exenteration surgery for advanced pelvic malignancy.^20^ This study found that pelvic exenteration patients experience an acute decline in post-operative function, and patients with a worse pre-operative function were more likely to experience worse post-operative morbidity.

Objective measurement of physical function in surgical patients is advantageous, especially when compared with patient-reported outcomes. In the pre-operative setting, functional tests can be used to determine fitness for surgery and predict post-operative outcomes.^21^ There is emerging evidence supporting the role of pre-operative optimisation through prehabilitation for improving physical fitness prior to surgery and increasing the likelihood of favourable post-operative outcomes.^22–26^ Post-operatively, physical function status can provide a gauge for recovery. On a population level, pre-operative and post-operative functional status of patients having CRS-HIPEC, and the association between pre-operative level of function and post-operative outcomes is poorly understood. Therefore, this prospective cohort study aims to describe the level of physical function in a CRS-HIPEC population using the 6MWT and 5STS, and to explore the association between pre-operative level of function and post-operative outcomes.

## Patients and Methods

### Study Design and Ethics

This is a prospective cohort study of patients who underwent CRS-HIPEC with curative intent for malignant disease at a single quaternary referral centre between 2017 and 2021.^27^ As part of the study, patients underwent assessment of physical function pre-operatively and at post-operative day 10. Ethics approval for this study was obtained from the Sydney Local Health District (Royal Prince Alfred Hospital Zone; Protocol No. X21-0236). Informed consent was obtained from all patients participating in the study. This study was conducted and written in accordance with the STROBE statement.^28^

### Study Variables

The primary outcomes of interest in this study were objective physical outcomes determined by 6MWT and 5STS (described below). These tests have been previously validated for assessing physical function in the setting of surgery, intensive care and chronic medical conditions.^20,29–32^ Functional assessments were performed pre-operatively and 10 days post-operatively by a registered physiotherapist. Pre-operative variables pertaining to patient characteristics included operative and admission details (including the type and extent of surgery performed, details of HIPEC delivery and completeness of cytoreduction or CC score), type of primary tumour, peritoneal cancer index (PCI),^5^ age, sex, height, weight, American Society of Anesthesiologists (ASA) physical status score, Eastern Cooperative Oncology Group (ECOG) performance status score, type of HIPEC agent, stoma type and discharge destination. Variables related to post-operative outcomes included length of hospital stay (LOS), intensive care unit (ICU) stay and post-operative in-hospital complications—described based on whether they were present or absent and according to the Comprehensive Complication Index (CCI) and Clavien–Dindo (CD) score.^33,34^ Patient characteristics and surgical outcomes were collected as part of a prospective research database using a standardized data collection form

### Six-Min Walk Test

The 6MWT assesses functional capacity and was conducted in accordance with the protocol of the American Thoracic Society.^26,35^ In brief, patients were instructed to walk as quickly as possible for 6 min up and down a 30 m straight, indoor corridor. The total distance walked (measured in metres) and patient vital signs (blood pressure, heart rate and oxygen saturation) were recorded. A higher 6-min walk distance (6MWD) indicates better functional capacity. All tests were performed in the same corridor. As per the ATS guidelines,^35^ the hospital physiotherapist performed the 6MWT once, with or without a prior practice test. If a practice test was performed, the second test was performed at least 1 h after the practice test, and the highest 6MWD was reported. Reference values for the 6MWT were derived from a mathematical model of healthy Australian participants:^36^

$$6{\text{MWD}} = 216.90 + 4.12 \times \left( {{\text{height}},{\text{cm}}} \right) - 1.75 \times \left( {{\text{age}},{\text{ years}}} \right) - 1.15 \times \left( {{\text{weight}},{\text{kg}}} \right) - 34.04 \times \left( {{\text{gender}},{\text{where}}\,{\text{men}} = 0\,{\text{and}}\,{\text{women}} = 1} \right).$$

These values were compared with the pre-operative 6MWD measured within the population of this study.

### Five-Times Sit to Stand Test

The 5STS assesses lower limb strength and function^26,37^ and was conducted as described previously.^20^ Patients were instructed to sit with arms folded across the chest and with their back against a chair. Patients were then asked to stand up and sit down as quickly as possible, five times. Time taken to complete the test was measured in seconds. A shorter time for completion of the 5STS is indicative of better physical function. All tests were performed in the same setting and used the same chair.

### Statistical Methods

Categorical data are expressed as frequency (percentage) and continuous data as median (interquartile range, IQR). Categorical variables were analysed using the Chi-squared test. Continuous variables were analysed using non-parametric tests such as the Wilcoxon test (for repeated measures analysis), Mann–Whitney *U* test (for unpaired column analysis) and Kruskal Wallis with Dunn’s multiple comparisons test (for column analysis involving three variables). Holm–Bonferroni post-hoc correction was added to column analysis to account for the number of variables and presented as a separate data set (Supplementary Fig. 2). Data obtained from 6MWT and 5STS were expressed as either absolute values of 6MWD (metres) or time taken to complete the 5STS (s), or as percentage change. The 6MWD percentage change was calculated using the following formula: [(pre-operative 6MWD—post-operative 6MWD)/pre-operative 6MWD]*100. The 5STS percentage change was calculated using the following formula: [(post-operative 5STS—pre-operative 5STS)/pre-operative 5STS]*100. A greater percentage change in 6MWD and 5STS represents a greater decline in physical function. Pearson’s correlation analysis was performed to determine correlations between 6MWT and 5STS outcomes, which were expressed as a *r* and *p*-values. An *r* value between 0 and ± 0.3 indicates a weak correlation, between 0.3 and 0.7 (or between − 0.3 and − 0.7) indicates a moderate correlation and between 0.7 and 1 (or between − 0.7 and − 1) indicates a strong correlation.^38^ For subgroup analysis of association between physical function and pre-operative and post-operative variables, variables were dichotomised based on their respective medians. All statistical calculations were performed on GraphPad Prism software (version 9). Statistical significance was set at *p* < 0.05 for all analyses.

## Results

### Characteristics of the Study Sample

**Fig. 1:**
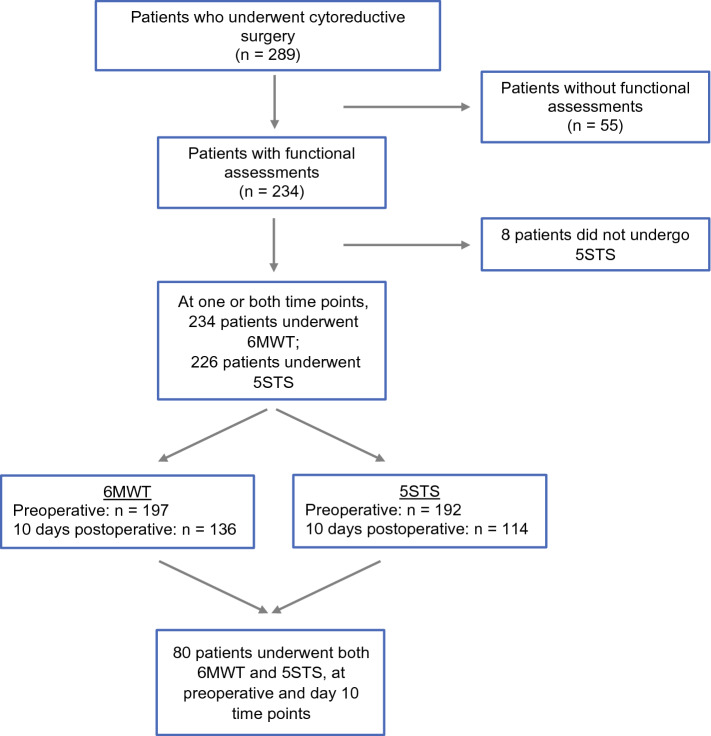
STROBE diagram describing the proportion of surgical patients who underwent physical function tests at pre-operative, day 10 post-operative or both time points. Reasons for patients not participating in functional tests include availability of hospital physiotherapist and patient discharge prior to day 10 time point

The study consisted of 289 consecutive patients who underwent CRS-HIPEC between April 2017 and July 2021. Of this cohort, 234 (81%) patients underwent at least one functional test at either pre-operative or at day 10 post-operative (Fig. 1). Eighty (34.2%) patients participated in both 6MWT and 5STS at both time points.

The median (IQR) age of patients who underwent physical function tests (*n* = 234) was 56 (20) years, and overall included more females (*n* = 124, 53%) than males (*n* = 110, 47%). The cohort consisted primarily of colorectal cancer patients (*n* = 107, 45.7%). The median PCI was 12, and 81.6% of patients underwent a complete cytoreduction with no residual peritoneal disease (CC-0). The median LOS was 18 days, and the median stay in ICU was 5 days. Most patients experienced post-operative complications (*n* = 174; 74.4%) and of these, the majority experienced minor complications [CD grade I–II (*n* = 119, 68.4%) or CCI ≤ 6 (*n* = 210; 89.7%)]. Characteristics of patients having CRS-HIPEC are detailed in Table 1. There were significant differences in ASA score and post-operative complications between patients who underwent and did not undergo physical function tests (Table 1).

**Table 1:** Characteristics of patients who underwent abdominal and pelvic cytoreductive surgery

	Patients with functional tests (*n* = 234)	Patients without functional tests (*n* = 55)	*p*-values
*Patient demographics*			
Age, years	56 (20) Range = 61	57 (20.3) Range = 57	0.88
*Sex*			**0.03**
Male	110 (47%)	17 (31%)	
Female	124 (53%)	38 (69%)	
BMI	27.9 (7.5) Range = 33.1	25 (7) Range = 23.1	**0.01**
*ASA score*			**< 0.001**
1	8 (3.4%)	3 (5.5%)	
2	104 (44.4%)	31 (56.4%)	
3	122 (52.1%)	18 (32.7%)	
4	0 (0%)	4 (7.3%)	
*ECOG score*			0.16
0	162 (69.2%)	40 (72.8%)	
1	58 (24.8%)	11 (20%)	
2	13 (5.6%)	2 (3.6%)	
3	1 (0.4%)	2 (3.6%)	
*Surgical factors*			
*Type of tumour*			0.61
Colorectal	107 (45.7%)	27 (49.1%)	
Appendix adenocarcinoma	49 (20.9%)	13 (23.6%)	
Ovarian	16 (6.8%)	3 (5.5%)	
Peritoneal mesothelioma	13 (5.6%)	2 (3.6%)	
Pseudomyxoma peritonei	40 (17.1%)	5 (9.1%)	
Small bowel adenocarcinoma	6 (2.6%)	0 (0%)	
Others	3 (1.3%)	5 (9.1%)	
Peritoneal cancer index (PCI)	12 (16) Range = 39	10 (21) Range = 37	0.63
*Peritonectomy*			0.99
Right parietal	226 (96.6%)	49 (89.1%)	
Left parietal	216 (92.3%)	47 (85.5%)	
Right subdiaphragmatic	137 (58.5%)	33 (60%)	
Left subdiaphragmatic	93 (39.7%)	24 (43.6%)	
Pelvic	190 (81.2%)	47 (85.5%)	
Right liver capsule	54 (23.1%)	14 (25.5%)	
Left liver capsule	32 (13.7%)	6 (10.9%)	
Stripping of porta hepatis	41 (17.5%)	11 (20%)	
*Completeness of cytoreduction*			0.93
CC-0	191 (81.6%)	45 (81.8%)	
CC-1	27 (11.5%)	7 (12.7%)	
CC-2	3 (1.3%)	1 (1.8%)	
CC-3	13 (5.6%)	2 (3.6%)	
*HIPEC*			**0.03**
Oxaliplatin	46 (19.7%)	2 (3.6%)	
Cisplatin	12 (5.1%)	4 (7.3%)	
Mitomycin-C	151 (64.5%)	44 (80%)	
Other	13 (5.6%)	0 (0%)	
None	12 (5.1%)	5 (9.1%)	
*Stoma*			**0.008**
Colostomy	15 (6.4%)	8 (14.5%)	
End ileostomy	23 (9.8%)	2 (3.6%)	
Defunctioning ileostomy	33 (14.1%)	15 (27.3%)	
None	163 (69.7%)	30 (54.6%)	
*Postoperative outcomes*			
Length of hospital stay, days	18 (11) Range = 153	19 (18) Range = 74	0.11
Intensive care unit stay, days	5 (2) Range = 75	5 (2) Range = 28	0.33
*Discharge destination*			0.66
Home	213 (91.1%)	49 (89.1%)	
Other hospital	5 (2.1%)	5 (9.1%)	
Rehabilitation	12 (5.1%)	0 (0%)	
Deceased in hospital	4 (1.7%)	1 (1.8%)	
*Number of hospital readmissions*			**0.03**
No readmissions	146 (62.4%)	43 (78.2%)	
≥ 1 readmissions	88 (37.6%)	12 (21.8%)	
*Post-operative complications*			**< 0.001**
Complications	174 (74.4%)	38 (69.1%)	
No Complications	59 (25.2%)	9 (16.4%)	
Missing data	1 (0.4%)	8 (14.5%)	
*Clavien–Dindo (n)*			0.21
I–II	119 (68.4%)	22 (57.9%)	
III–V	55 (31.6%)	16 (42.1%)	
*Comprehensive Complication Index (CCI)*			0.89
≤ 6	210 (89.7%)	49 (89.1%)	
> 6	24 (10.3%)	6 (10.9%)	

Categorical variables presented as frequency (percentage) and were analysed using Chi-squared test. Continuous variables presented as median, interquartile range (IQR) and range, and were analysed used Mann–Whitney *U* test. Statistical significance is set at *p* < 0.05. Significant *p*-values are represented in bold

### Pre-operative and Post-operative Physical Function

One hundred ninety-seven (84.2%) patients underwent the 6WMT pre-operatively, 136 (58.1%) on post-operative day 10 and 99 (42.3%) patients at both time points. One hundred ninety-two (82.1%) patients underwent the 5STS pre-operatively, 114 (48.7%) patients on post-operative day 10 and 80 (34.2%) at both time points (Fig. 1).

**Fig. 2:**
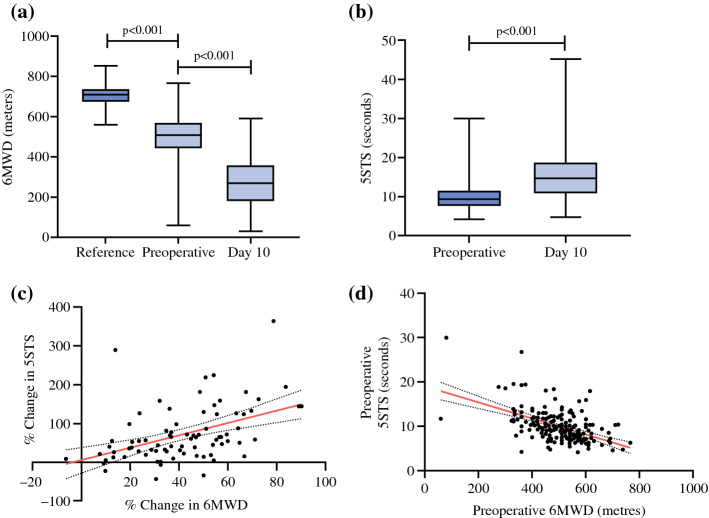
Outcomes of functional assessments in patients who have undergone cytoreductive surgery (CRS), performed at pre-operative and post-operative day 10 time points. **a** Comparison of 6-min walk distance (6MWD) among reference (*n* = 197), pre-operative (*n* = 197) and post-operative day 10 (*n* = 136). A higher 6MWD value denotes better function. Reference was derived from a mathematical model of healthy participants. **b** Comparison of five-times sit to stand (5STS) between pre-operative (*n* = 192) and post-operative day 10 (*n* = 114). A higher 5STS value denotes worse function. **c** Correlation analysis comparing the percentage change in 6MWD (*n* = 80) to percentage change in 5STS (*n* = 80) following surgery. **d** Preoperative 6MWD (*n* = 197) to 5STS (*n* = 192) using correlation analysis. Column analysis was performed using the Wilcoxon or Mann–Whitney *U* tests and data is presented as median and 25th and 75th quartiles (error bars represent minimum and maximum values). Correlation analysis was performed using the Spearman test and plotted with a line of best fit and 95% confidence interval. Statistical significance is set at *p* < 0.05

Pre-operatively, patients were functionally impaired compared with the general Australian population^36^ [pre-operative 6MWD median (IQR): 510 m (127 m); reference 6MWD: 709.7 m (65.5 m); *p* < 0.001] (Fig. 2a). At post-operative day 10, patients experienced a further decline in 6MWD compared with pre-operative levels [post-operative 6MWD: 270 m (180 m); *p* < 0.001]. Post-operatively, patients also showed impairment in the 5STS performance [pre-operative 5STS median (IQR): 9.3 s (4.1 s); post-operative 5STS: 14.7 s (8.1 s); *p* < 0.001] (Fig. 2b). The percentage change in 6MWD showed a moderate positive linear correlation with the percentage change in 5STS (*r* = 0.51; *p* < 0.001) (Fig. 2c). Similarly, pre-operative 6MWD showed a moderate negative linear correlation with pre-operative 5STS (*r* = − s0.48; *p* < 0.001) (Fig. 2d).

**Fig. 3:**
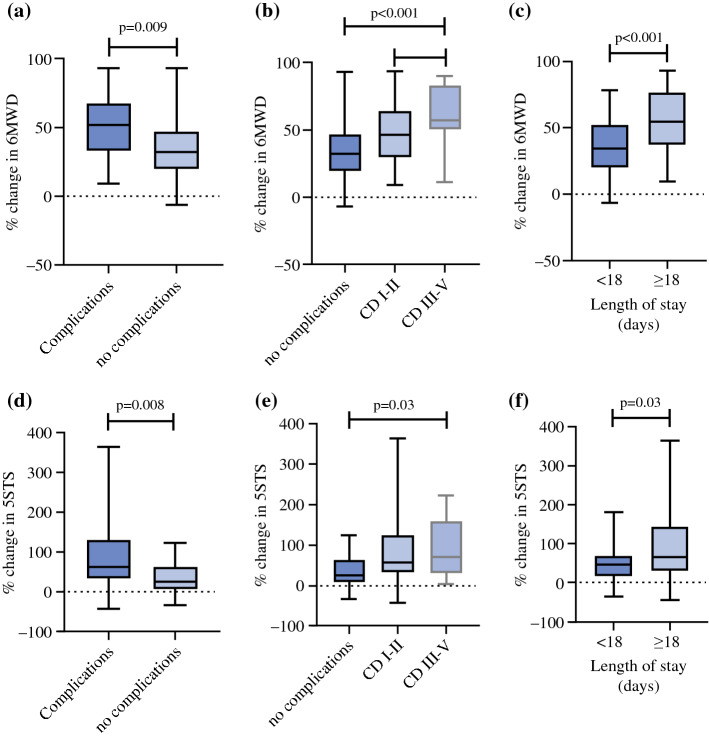
Association between percentage change in physical function, and post-operative outcomes. Percentage change in function was calculated for 6-min walk distance (6MWD) with the following formula: [(pre-operative 6MWD – postperative 6MWD)/pre-operative 6MWD]*100. Percentage change in function was calculated for five-times sit to stand test (5STS) with the following formula: [(postperative 5STS—pre-operative 5STS)/pre-operative 5STS]*100. **a**–**c** percentage change in 6MWD, **d**–**f** percentage change in 5STS, analysed based on complications [occurrence, Clavien–Dindo (CD)] and length of hospital stay (LOS). Continuous variables were dichotomised based on the median value. Data is presented as median and interquartile range (error bars show minimum and maximum). Statistical significance is determined using the Mann–Whitney *U* test or Kruskal Wallis with Dunn’s multiple comparisons test. Significance is set at *p* < 0.05

Differences in 6MWD and 5STS percentage change were assessed in a subgroup analysis according to patient pre-operative and peri-operative characteristics (Supplementary Table 1). There were no differences in the percentage change of 6MWD and 5STS based on age, sex, body mass index (BMI), ASA and ECOG score. Peri-operatively, a higher cancer burden (PCI ≥ 12) and incomplete cytoreduction was associated with a greater post-operative decline in function, with no significant difference in patients receiving mitomycin-C compared with platinum-based HIPEC. Post-operatively, greater severity of post-operative complications (CD grade III–V) and longer LOS (≥ 18 days) were associated with a greater post-operative decline in function (Fig. 3). There were no differences in ICU stay or number of hospital readmissions (Supplementary Fig. 1)

### Pre-operative Function and Post-operative Outcomes

Associations between pre-operative functional capacity and post-operative outcomes are detailed in Table 2. Pre-operatively, patients with a higher level of pre-operative function were more likely to be younger (< 56 years), male, have lower ASA and ECOG scores, have a lower cancer burden (PCI < 12) and be candidates for complete cytoreduction. Post-operatively, patients with better pre-operative function were more likely to have a shorter LOS [6MWD <18 days: 530 (96.5); ≥ 18 days: 471 (145.8); *p* < 0.001: 5STS <18 days: 8.89 (3); ≥ 18 days: 10.21 (4.4); *p* < 0.03] and minor post-operative complications, i.e. CD [6MWD grade I–II: 525 (135); grade III–V: 475 (112.3); *p* = 0.01] and CCI [6MWD CCI ≤ 6: 510 (121.5); CCI > 6: 460 (95); *p* = 0.04: 5STS CCI ≤ 6: 9.18 (4.1); CCI > 6: 11.01 (3.6); *p* = 0.01]. They were also more likely to be discharged home [6MWD: 510 (120): 5STS: 9.17 (3.9)] as opposed to being transferred to another hospital or to in-patient rehabilitation, or dying [6MWD: 424 (155.5); *p* = 0.005: 5STS: 11.61 (5.05); *p* < 0.001].

**Table 2:** Association between pre-operative functional capacity and pre-operative patient characteristics and post-operative outcomes

Patient characteristics	Pre-operative 6MWD (m) (*n* = 197)		Pre-operative 5STS (s) (*n* = 192)	
*Age (years)*				
< 56	*N* = 95	540 (116)	*N* = 94	8.49 (3.3)
≥ 56	*N* = 102	487 (105.8)	*N* = 98	10.26 (3.44)
*p-*Value	**0.004**		**< 0.001**	
*Sex*				
Male	*N* = 95	515 (110)	*N* = 90	9.25 (3.8)
Female	*N* = 102	488 (146.2)	*N* = 102	9.4 (4.3)
*p*-value	**0.04**		0.81	
*BMI*				
< 27.9	*N* = 102	510 (120)	*N* = 99	8.90 (4.0)
≥ 27.9	*N* = 95	505 (123)	*N* = 93	9.43 (4.1)
*p*-value	0.24		0.17	
*ASA score*				
*1*	*N* = 8	568 (183)	*N* = 6	8.45 (4.0)
*2*	*N* = 81	540 (96)	*N* = 79	8.65 (3.2)
*3*	*N* = 108	475 (138.5)	*N* = 107	10.19 (3.7)
*p*-value	1 versus 2: 0.68		1 versus 2: 0.99	
	1 versus 3: **0.009**		1 versus 3: 0.70	
	2 versus 3: **< 0.001**		2 versus 3: **0.006**	
*ECOG score*				
0	*N* = 133	540 (99)	*N* = 129	8.87 (3.1)
1	*N* = 51	440 (133)	*N* = 50	10.83 (3.3)
2	*N* = 13	400 (153.5)	*N* = 13	13.80 (9.3)
*p*-value	0 versus 1: **< 0.001**		0 versus 1: **0.007**	
	0 versus 2: **< 0.001**		0 versus 2: **< 0.001**	
	1 versus 2: 0.26		1 versus 2: 019	
*Peritoneal cancer index (PCI)*				
< 12	*N* = 94	527.5 (115.8)	*N* = 91	9.15 (3.8)
≥ 12	*N* = 103	500 (125)	*N* = 101	9.60 (4.0)
*p*-value	**0.006**		0.12	
*Completeness of cytoreduction*				
CC-0	*N* = 157	510 (120)	*N* = 153	9.15 (3.9)
CC-1, 2 and 3	*N* = 40	475 (134.8)	*N* = 39	10.35 (3.0)
*p*-values	**0.04**		**0.03**	
*Length of hospital stay (days)*				
< 18	*N* = 93	530 (96.5)	*N* = 90	8.89 (3.0)
≥ 18	*N* = 104	471 (145.8)	*N* = 102	10.21 (4.4)
*p*-value	**< 0.001**		**0.03**	
*Intensive care unit stay (days)*				
< 5	*N* = 84	521.5 (102.3)	*N* = 79	8.99 (2.9)
≥ 5	*N* = 113	500 (150)	*N* = 113	9.81 (4.52)
*p*-value	0.07		0.21	
*Number of hospital readmissions*				
0	*N* = 114	523.5 (120)	*N* = 113	8.9 (3.9)
≥ 1	*N* = 83	490 (120)	*N* = 79	10.19 (4.2)
*p*-value	0.07		0.07	
*Discharge destination*				
Home	*N* = 176	510 (120)	*N* = 171	9.17 (3.9)
Other (other hospital, rehabilitation, deceased in hospital)	*N* = 21	424 (155.5)	*N* = 21	11.61 (5.05)
*p*-value	**0.005**		**< 0.001**	
*Post-operative complications*				
Complications	*N* = 139	510 (135)	*N* = 137	9.17 (4.2)
No complications	*N* = 57	510 (105)	*N* = 54	9.96 (3.8)
*p*-value	0.81		0.53	
*Clavien–Dindo*				
I–II	*N* = 91	525 (120)	*N* = 90	8.90 (3.8)
III–V	*N* = 48	475 (112.3)	*N* = 47	9.81 (5.3)
*p*-value	**0.01**		0.06	
*Comprehensive Complication Index (CCI)*				
≤ 6	*N* = 176	510 (121.5)	*N* = 172	9.18 (4.1)
> 6	*N* = 21	460 (95)	*N* = 20	11.01 (3.6)
*p*-value	**0.04**		**0.01**	

Categorical variables presented as frequency (number of patients). Continuous variables presented as median and interquartile range (IQR) and were analysed used Mann–Whitney *U* test or Kruskal–Wallis test with Dunn’s multiple comparisons. Statistical significance is set at *p* < 0.05. Significant *p*-values are shown in bold

The association between pre-operative function and post-operative surgical outcomes were further explored by dichotomising both pre-operative 6MWD and 5STS time by the cohort median (i.e. 6MWD: 510 m and 5STS: 9.3 s) (Supplementary Table 2), and the results were comparable to the analysis detailed in Table 2. Patients with pre-operative 6MWD ≥ 510 m and 5STS < 9.3 s had a shorter LOS, fewer number of hospital readmissions and minor post-operative complications (CD grade I–II and CCI ≤ 6).

## Discussion

### Summary of Findings

The main findings of this CRS-HIPEC study suggest that patients with a higher level of pre-operative physical function were more likely to have a shorter LOS, experience more minor post-operative complications (grade I–II) and were more likely to be discharged home. However, pre-operative physical function was not associated with ICU stay, number of hospital readmissions and the presence (as opposed to absence) of post-operative complications. Patients experience a further decline in physical function after surgery, and a greater decline in physical function was associated with a higher peritoneal metastasis burden, incomplete cytoreduction, presence of post-operative complication, major post-operative complications (grade III–V) and longer LOS.

### Physical Status of CRS-HIPEC Patient Population

A decline in pre-operative function in comparison with the general population is similar to outcomes previously reported in other major cancer patients.^20^ Notably, this study found that patients with worse pre-operative function were more likely to experience major post-operative complications. Several studies in gastrointestinal, abdominal and pelvic onco-surgeries have found that poorer pre-operative functional capacity is associated with increased post-operative morbidity.^16–19^ In CRS-HIPEC, Pillinger et al.^39^ reported that patients who did not develop post-operative complications had a higher pre-operative peak VO_2_ on cardiopulmonary exercise testing (CPET). A recent review identified several modifiable and non-modifiable pre-operative risk factors such as age, sex, ASA score, ECOG performance, QoL and nutrition that can determine post-operative complications, LOS and survival after CRS-HIPEC.^9^ This study establishes pre-operative physical function as a potentially modifiable risk factor impacting post-operative morbidity, which may be optimised through physical rehabilitation prior to surgery. However, pre-operative function was not a significant predictor of hospital readmission in this study, which suggests that there may be other factors impacting post-operative recovery. There is increasing evidence to support the role of prehabilitation for pre-operative optimisation in oncological surgery.^23,24^ Whilst evidence supporting prehabilitation in the CRS-HIPEC patient population is scarce,^9^ recommendations supporting prehabilitation in this population have been published.^40^

Following surgery, patients having CRS-HIPEC experience a further decline in physical function with respect to their pre-operative baseline, with a greater decline being associated with greater post-operative morbidity. This has also been reported in patients who have undergone pelvic exenteration,^20^ and agrees with patient-reported QoL outcomes which also demonstrate a decline in QoL measures in the acute post-operative period, and an association with post-operative morbidity.^10–13,41^ These studies also report a long-term recovery of QoL. Whether recovery of physical function follows a similar trajectory as QoL remains to be studied.

The utility of the 6MWT and 5STS in assessing physical function in surgical and chronically ill patients has been previously demonstrated.^29–32,42,43^ The 6MWT offers a relatively inexpensive, less labour intensive and widely applicable tool for measuring functional capacity of patients in the clinical setting, compared with CPET, which is the current gold standard. Comparison between CPET and 6MWD and their correlation with post-operative outcomes is somewhat limited and warrants investigation. The 5STS is a validated tool for assessing functional lower extremity strength, balance and exercise capacity^37,44,45^ that is moderately correlated with 6MWT in the CRS-HIPEC patient population, as demonstrated in this study. Several studies have demonstrated the clinical utility of these tests in assessing physical function following abdominal surgery, with poorer function being associated with worse post-operative morbidity.^17,20,46,47^ Given that both tests measure different aspects of physical function and only show moderate correlation, they should not be used interchangeably. Also, both the 6MWT and 5STS have only been validated in face-to-face encounters and their application in telehealth is yet to be examined. However, it is expected that the 5STS will be easier to administer through the telehealth interface.

### Limitations

Aspects of patient demographics may have confounded this study. Despite this study including a large sample, several differences in patient characteristics were found between patients who participated compared with those who did not participate in physical function tests, which may have introduced bias to this study. Moreover, this is a single centre study, which arguably restricts the generalisability of the conclusions derived from this study. A multi-centre study assessing the association between physical function and surgical outcomes would provide more meaningful conclusions and may be more impactful in informing international clinical guidelines. This study is also limited in that fewer than 40% of patients participated in both 6MWT and 5STS at both the pre-operative and day 10 post-operative time points. Patient participation was primarily impacted by logistical factors such as competing demands of the clinical team, time pressure on clinics and the availability of hospital physiotherapists to implement functional assessments owing to staff shortages. A minority of patients had a prolonged ICU stay exceeding 10 days and so these patients did not participate in functional assessment on post-operative day 10. Post-operative functional outcomes may also be confounded by acute post-operative pain, which was not measured in this study. This study could also be improved by examining the impact of factors such nutritional status, extent of the procedure and pre-operative comorbidities on performance in pre-operative and post-operative functional assessment.

## Conclusions

Cancer patients who are candidates for CRS-HIPEC have worse pre-operative functional capacity compared with the general Australian population. Poorer pre-operative function is associated with a longer LOS and major post-operative complications. Post-operatively, patients undergoing CRS-HIPEC experience a further decline in physical function, with a greater extent of decline being associated with higher cancer burden, incidence and severity of post-operative complications and longer LOS. This study is the first of its kind to quantify pre-operative and acute post-operative physical function in the CRS-HIPEC patient population using objective measures, and to provide evidence for an association between pre-operative function and post-operative outcomes in this population. This study has important implications for improving clinical decision-making, patient education and for establishing strategies to improve pre-operative function for mitigating post-operative morbidity. Future studies investigating the long-term trajectories of physical function, including at time of discharge and at various post-discharge time points, are warranted, which can be correlated with QoL outcomes. Examining other pre-operative variables in conjunction with physical function such as nutritional and psychological statuses, and the extent of cancer-induced sarcopenia will also provide important information. The association of physical function and post-operative outcomes should be further elucidated in a multivariate analysis using broader known predictive factors. This would lend support for future randomised controlled trials investigating the role of prehabilitation for improving post-operative outcomes in the CRS-HIPEC patient population.

## Supplementary Information

Below is the link to the electronic supplementary material.

Supplementary file1 (DOCX 225 kb)

Supplementary file2 (DOCX 251 kb)

Supplementary file3 (DOCX 21 kb)

Supplementary file4 (DOCX 28 kb)
