# Supplementary material for: Functional Outcomes Following Cytoreductive Surgery and Hyperthermic Intraperitoneal Chemotherapy: A Prospective Cohort Study
Source: Ann Surg Oncol. 2022 Oct 28;30(1):447–58. doi: 10.1245/s10434-022-12691-x (PMC9726807; doi:10.1245/s10434-022-12691-x)
Supplement: Supplementary file 1 — Supplementary file1 (DOCX 225 kb) [file 10434_2022_12691_MOESM1_ESM.docx]

| 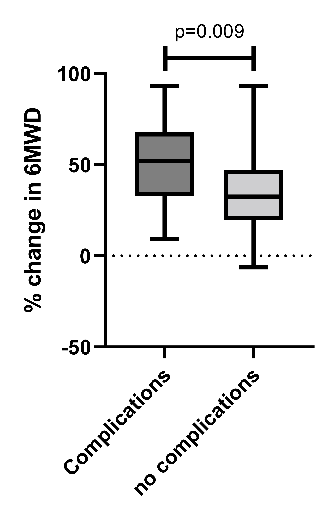  **A**  **B**  **F** | 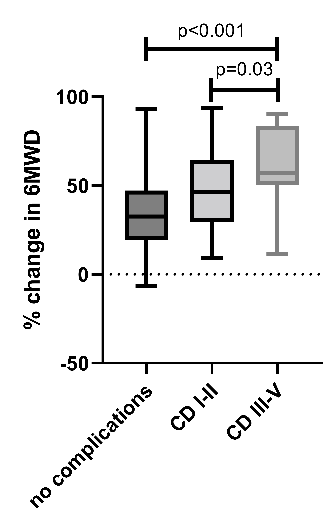  **E**  **G** | 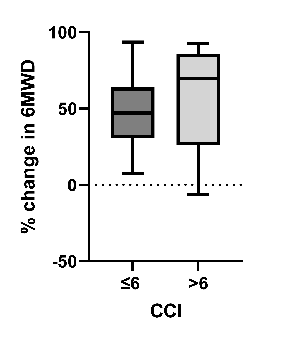  **D**  **C**  **H** | 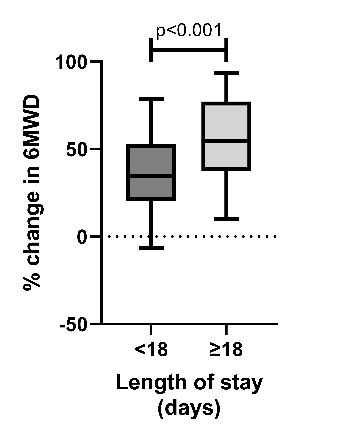 |
| --- | --- | --- | --- |
| 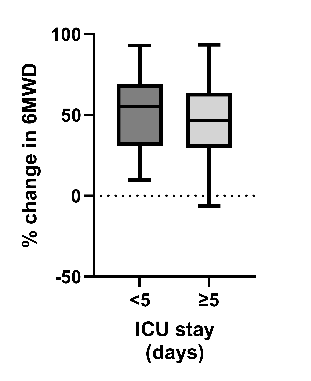  **I**  **J** | 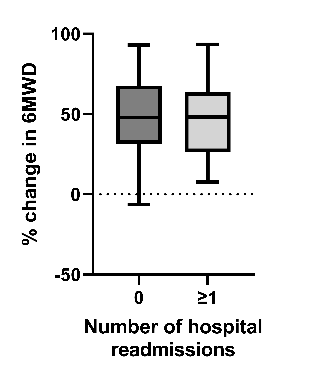  **K** | 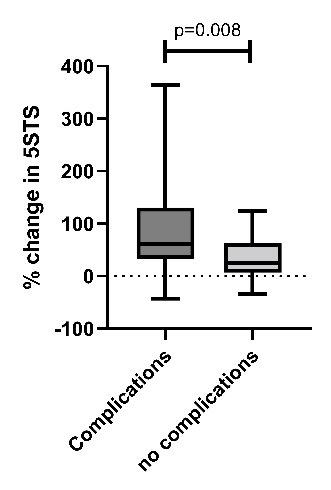  **L** | 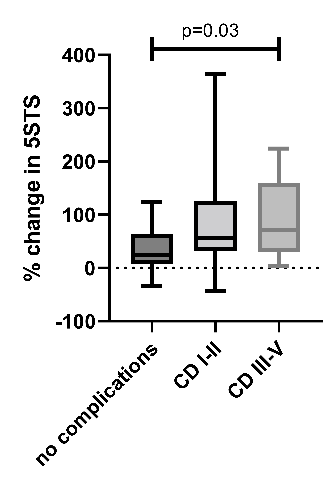 |
| 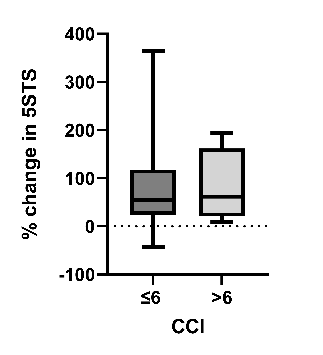 | 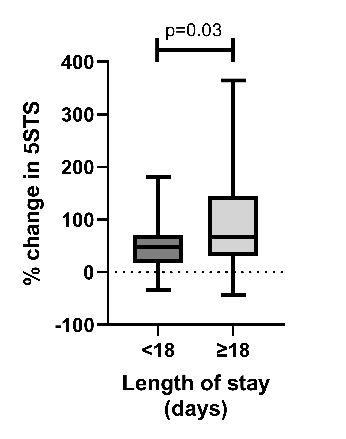 | 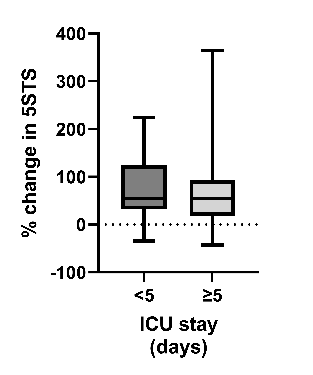 | 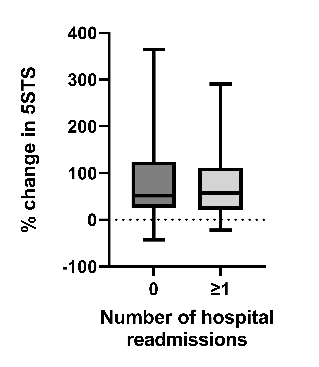 |
| **Supplementary Figure 1:**  Association between percentage change in physical function, and postoperative outcomes, analysed with Holm-Bonferroni post-hoc correction. Percentage change in function was calculated for six-minute walk distance (6MWD) with the following formula: [(preoperative 6MWD – postperative 6MWD)/preoperative 6MWD]*100. Percentage change in function was calculated for five-times sit to stand test (5STS) with the following formula: [(postperative 5STS – preoperative 5STS)/preoperative 5STS]*100. Graphs A to F show percentage change in 6MWD, and graphs D to F represent percentage change in 5STS, analysed based on complications (occurrence, Clavien Dindo (CD) and Comprehensive Complication Index (CCI)), length of hospital stay (LOS), ICU stay and number of hospital readmissions. Continuous variables were dichotomised based on the median value. Data is presented as median and interquartile range (error bars show minimum and maximum). Statistical significance is determined using the Mann-Whitney test with Holm-Bonferonni post-hoc correction or Kruskal Wallis with Dunn’s multiple comparisons test. Significance is set at p<0.05. | | | |

**E**

**D**

**C**

**B**

**A**
