# Supplementary material for: Functional Outcomes Following Cytoreductive Surgery and Hyperthermic Intraperitoneal Chemotherapy: A Prospective Cohort Study
Source: Ann Surg Oncol. 2022 Oct 28;30(1):447–58. doi: 10.1245/s10434-022-12691-x (PMC9726807; doi:10.1245/s10434-022-12691-x)
Supplement: Supplementary file 2 — Supplementary file2 (DOCX 251 kb) [file 10434_2022_12691_MOESM2_ESM.docx]

**Supplementary Figure 2 –** Holm-Bonferroni post-hoc correction added to column analyses. Tables and figures re-presented with this correction.

| Characteristics of patients who underwent abdominal and pelvic cytoreductive surgery – with Holm-Bonferroni post-hoc correction | | | |
| --- | --- | --- | --- |
|  | **Patients with functional tests**  **(n = 234)** | **Patients without functional tests**  **(n = 55)** | **p-values** |
| *Patient demographics* | | | |
| Age, years | 56 [20]  *range=61* | 57 [20.3]  *range=57* | 1.0 |
|  |  |  |  |
| Sex |  |  | 0.24 |
| *Male* | 110 (47%) | 17 (31%) |  |
| *Female* | 124 (53%) | 38 (69%) |  |
| BMI | 27.9 [7.5]  *range=33.1* | 25 [7]  *range=23.1* | 0.09 |
|  |  |  |  |
| ASA score |  |  | **0.001** |
| *1* | 8 (3.4%) | 3 (5.5%) |  |
| *2* | 104 (44.4%) | 31 (56.4%) |  |
| *3* | 122 (52.1%) | 18 (32.7%) |  |
| *4* | 0 (0%) | 4 (7.3%) |  |
|  |  |  |  |
| ECOG score |  |  | 0.96 |
| *0* | 162 (69.2%) | 40 (72.8%) |  |
| *1* | 58 (24.8%) | 11 (20%) |  |
| *2* | 13 (5.6%) | 2 (3.6%) |  |
| *3* | 1 (0.4%) | 2 (3.6%) |  |
| *Surgical factors* | | | |
| Type of tumour |  |  | 1.0 |
| *Colorectal* | 107 (45.7%) | 27 (49.1%) |  |
| *Appendix adenocarcinoma* | 49 (20.9%) | 13 (23.6%) |  |
| *Ovarian* | 16 (6.8%) | 3 (5.5%) |  |
| *Peritoneal mesothelioma* | 13 (5.6%) | 2 (3.6%) |  |
| *Pseudomyxoma peritonei* | 40 (17.1%) | 5 (9.1%) |  |
| *Small bowel adenocarcinoma* | 6 (2.6%) | 0 (0%) |  |
| *Others* | 3 (1.3%) | 5 (9.1%) |  |
|  |  |  |  |
| Peritoneal Cancer Index (PCI) | 12 [16]  *range=39* | 10 [21]  *range=37* | 1.0 |
|  |  |  |  |
| Peritonectomy |  |  | 1.0 |
| *Right parietal* | 226 (96.6%) | 49 (89.1%) |  |
| *Left parietal* | 216 (92.3%) | 47 (85.5%) |  |
| *Right subdiaphragmatic* | 137 (58.5%) | 33 (60%) |  |
| *Left subdiaphragmatic* | 93 (39.7%) | 24 (43.6%) |  |
| *Pelvic* | 190 (81.2%) | 47 (85.5%) |  |
| *Right liver capsule* | 54 (23.1%) | 14 (25.5%) |  |
| *Left liver capsule* | 32 (13.7%) | 6 (10.9%) |  |
| *Stripping of porta hepatis* | 41 (17.5%) | 11 (20%) |  |
|  |  |  |  |
| Completeness of cytoreduction |  |  | 1.0 |
| *CC-0* | 191 (81.6%) | 45 (81.8%) |  |
| *CC-1* | 27 (11.5%) | 7 (12.7%) |  |
| *CC-2* | 3 (1.3%) | 1 (1.8%) |  |
| *CC-3* | 13 (5.6%) | 2 (3.6%) |  |
|  |  |  |  |
| HIPEC |  |  | 0.24 |
| *Oxaliplatin* | 46 (19.7%) | 2 (3.6%) |  |
| *Cisplatin* | 12 (5.1%) | 4 (7.3%) |  |
| *Mitomycin-C* | 151 (64.5%) | 44 (80%) |  |
| *Other* | 13 (5.6%) | 0 (0%) |  |
| *None* | 12 (5.1%) | 5 (9.1%) |  |
|  |  |  |  |
| Stoma |  |  | 0.08 |
| *Colostomy* | 15 (6.4%) | 8 (14.5%) |  |
| *End ileostomy* | 23 (9.8%) | 2 (3.6%) |  |
| *Defunctioning Ileostomy* | 33 (14.1%) | 15 (27.3%) |  |
| *None* | 163 (69.7%) | 30 (54.6%) |  |
| *Postoperative outcomes* | | | |
| Length of hospital stay, days | 18 [11]  *range=153* | 19 [18]  *range=74* | 0.55 |
|  |  |  |  |
| Intensive care unit stay, days | 5 [2]  *range=75* | 5 [2]  *range=28* | 0.99 |
|  |  |  |  |
| Discharge destination |  |  | 1.0 |
| *Home* | 213 (91.1%) | 49 (89.1%) |  |
| *Other hospital* | 5 (2.1%) | 5 (9.1%) |  |
| *Rehabilitation* | 12 (5.1%) | 0 (0%) |  |
| *Deceased in hospital* | 4 (1.7%) | 1 (1.8%) |  |
|  |  |  |  |
| Number of hospital readmissions |  |  | 0.18 |
| *No readmissions* | 146 (62.4%) | 43 (78.2%) |  |
| *≥1 readmissions* | 88 (37.6%) | 12 (21.8%) |  |
|  |  |  |  |
| Postoperative complications |  |  | **<0.001** |
| *Complications* | 174 (74.4%) | 38 (69.1%) |  |
| *No Complications* | 59 (25.2%) | 9 (16.4%) |  |
| *Missing data* | 1 (0.4%) | 8 (14.5%) |  |
|  |  |  |  |
| Clavien Dindo (n) |  |  | 0.84 |
| *I-II* | 119 (68.4%) | 22 (57.9%) |  |
| *III-V* | 55 (31.6%) | 16 (42.1%) |  |
|  |  |  |  |
| Comprehensive Complication Index (CCI) |  |  | 1.0 |
| *≤*6 | 210 (89.7%) | 49 (89.1%) |  |
| *>6* | 24 (10.3%) | 6 (10.9%) |  |
| *Categorical variables presented as frequency (percentage) and were analysed using Chi-squared test. Continuous variables presented as median, interquartile range [IQR] and range, and were analysed used Mann-Whitney test. Holm-Bonferroni test was used to adjust for multiple testing. Statistical significance is set at p<0.05. Significant p values are represented in bold.* | | | |

| Association between preoperative functional capacity, and preoperative patient characteristics and postoperative outcomes – with Holm-Bonferroni post-hoc correction | | | | |
| --- | --- | --- | --- | --- |
| **Patient characteristics** | **Preoperative 6MWD (metres)**  **(n = 197)** | | **Preoperative 5STS (seconds)**  **(n = 192)** | |
|  |  | |  | |
| Age (years) |  | |  | |
| *<56* | N = 95 | 540 [116] | N = 94 | 8.49 [3.3] |
| *≥56* | N = 102 | 487 [105.8] | N = 98 | 10.26 [3.44] |
| *p-values* | **0.02** | | **<0.001** | |
|  |  | |  | |
| Sex |  | |  | |
| *Male* | N = 95 | 515 [110] | N = 90 | 9.25 [3.8] |
| *Female* | N = 102 | 488 [146.2] | N = 102 | 9.4 [4.3] |
| *p-values* | 0.12 | | 0.81 | |
|  |  | |  | |
| BMI |  | |  | |
| *<27.9* | N = 102 | 510 [120] | N = 99 | 8.90 [4.0] |
| *≥27.9* | N = 95 | 505 [123] | N = 93 | 9.43 [4.1] |
| *p-values* | 0.24 | | 0.36 | |
|  |  | |  | |
| ASA score |  | |  | |
| *1* | N = 8 | 568 [183] | N = 6 | 8.45 [4.0] |
| *2* | N = 81 | 540 [96] | N = 79 | 8.65 [3.2] |
| *3* | N = 108 | 475 [138.5] | N = 107 | 10.19 [3.7] |
| *p-value* | 1 vs 2: 0.68  1 vs 3: **0.009**  2 vs 3: **<0.001** | | 1 vs 2: 0.99  1 vs 3: 0.70  2 vs 3: **0.006** | |
|  |  | |  | |
| ECOG score |  | |  | |
| *0* | N = 133 | 540 [99] | N = 129 | 8.87 [3.1] |
| *1* | N = 51 | 440 [133] | N = 50 | 10.83 [3.3] |
| *2* | N = 13 | 400 [153.5] | N = 13 | 13.80 [9.3] |
| *p-value* | 0 vs 1: **<0.001**  0 vs 2: **<0.001**  1 vs 2: 0.26 | | 0 vs 1: **0.007**  0 vs 2: **<0.001**  1 vs 2: 019 | |
|  |  | |  | |
| Peritoneal cancer index (PCI) |  | |  | |
| *<12* | N = 94 | 527.5 [115.8] | N = 91 | 9.15 [3.8] |
| *≥12* | N = 103 | 500 [125] | N = 101 | 9.60 [4.0] |
| *p-value* | **0.02** | | 0.36 | |
|  |  | |  | |
| Completeness of cytoreduction |  | |  | |
| *CC-0* | N = 157 | 510 [120] | N = 153 | 9.15 [3.9] |
| *CC-1, 2 & 3* | N = 40 | 475 [134.8] | N = 39 | 10.35 [3.0] |
| *p-value* | 0.12 | | 0.12 | |
|  |  | |  | |
| Length of hospital stay (days) |  | |  | |
| *<18* | N = 93 | 530 [96.5] | N = 90 | 8.89 [3.0] |
| *≥18* | N = 104 | 471 [145.8] | N = 102 | 10.21 [4.4] |
| *p-value* | **<0.001** | | 0.15 | |
|  |  | |  | |
| Intensive care unit stay (days) |  | |  | |
| *<5* | N = 84 | 521.5 [102.3] | N = 79 | 8.99 [2.9] |
| *≥5* | N = 113 | 500 [150] | N = 113 | 9.81 [4.52] |
| *p-value* | 0.21 | | 0.42 | |
|  |  | |  | |
| Number of hospital readmissions |  | |  | |
| *0* | N = 114 | 523.5 [120] | N = 113 | 8.9 [3.9] |
| *≥1* | N = 83 | 490 [120] | N = 79 | 10.19 [4.2] |
| *p-value* | 0.21 | | 0.24 | |
|  |  | |  | |
| Discharge destination |  | |  | |
| *Home* | N = 176 | 510 [120] | N = 171 | 9.17 [3.9] |
| *Other (other hospital, rehabilitation, deceased in hospital)* | N = 21 | 424 [155.5] | N = 21 | 11.61 [5.05] |
| *p-value* | **0.03** | | **0.003** | |
|  |  | |  | |
| Postoperative complications |  | |  | |
| *Complications* | N = 139 | 510 [135] | N = 137 | 9.17 [4.2] |
| *No complications* | N = 57 | 510 [105] | N = 54 | 9.96 [3.8] |
| *p-value* | 0.81 | | 0.53 | |
| Clavien-Dindo |  | |  | |
| *I-II* | N = 91 | 525 [120] | N = 90 | 8.90 [3.8] |
| *III-V* | N = 48 | 475 [112.3] | N = 47 | 9.81 [5.3] |
| *p-value* | 0.05 | | 0.24 | |
|  |  | |  | |
| Comprehensive Complication Index (CCI) |  | |  | |
| *≤6* | N = 176 | 510 [121.5] | N = 172 | 9.18 [4.1] |
| *>6* | N = 21 | 460 [95] | N = 20 | 11.01 [3.6] |
| *p-value* | 0.16 | | 0.06 | |
| *Categorical variables presented as frequency (percentage) and were analysed using Chi-squared test. Continuous variables presented as median, interquartile range [IQR] and range, and were analysed used Mann-Whitney test. Holm-Bonferroni test was used to adjust for multiple testing. Statistical significance is set at p<0.05. Significant p values are represented in bold.* | | | | |

| Percentage change in physical function following cytoreductive surgery, based on patient characteristics, oncological and surgical factors - with Holm-Bonferroni post-hoc correction | | | | |
| --- | --- | --- | --- | --- |
| **Preoperative patient characteristics** | **% Change in 6MWD**  **(n = 99)** | | **% Change in 5STS**  **(n = 80)** | |
| Age (years) |  | |  | |
| *<56* | N = 50 | 47.7% [34.6%]] | N = 44 | 48% [110.7%] |
| *≥56* | N = 49 | 48.1% [36.5%] | N = 36 | 57.1% [63.6%] |
| *p-values* | 1.00 | | 1.00 | |
|  |  | |  | |
| Sex |  | |  | |
| *Male* | N = 44 | 36.8% [39.8%] | N = 34 | 49.9% [55.7%] |
| *Female* | N = 55 | 53% [30.6%] | N = 46 | 59.3% [109.4%] |
| *p-values* | 0.42 | | 1.00 | |
|  |  | |  | |
| BMI |  | |  | |
| *<27.9* | N = 52 | 47.7% [32.4%] | N = 43 | 49.7% [48.8%] |
| *≥27.9* | N = 47 | 48.6% [37.7%] | N = 37 | 64.8% [108%] |
| *p-values* | 1.00 | | 1.00 | |
|  |  | |  | |
| ASA score |  | |  | |
| *1* | N = 2 | 53.1% [12.7%] | N = 2 | 67.8% [7.6%] |
| *2* | N = 41 | 47.2% [38.1%] | N = 36 | 54.5% [92.9%] |
| *3* | N = 56 | 48.3% [36%] | N = 42 | 49.8% [73%] |
| *p-value* | 0.65 | | 0.30 | |
|  |  | |  | |
| ECOG score |  | |  | |
| *0* | N = 67 | 45.9% [31.2%] | N = 57 | 51.4% [67.9%] |
| *1* | N = 29 | 50% [36.4%] | N = 22 | 66.7% [103.2%] |
| *2* | N = 3 | 82.2% [32%] | N = 1 | 4.2% [0%] |
| *p-value* | 0.14 | | 0.28 | |
|  |  | |  | |
| Peritoneal cancer index  (PCI) |  | |  | |
| *<12* | N = 43 | 36.8% [33.9%] | N = 38 | 48.2% [67%] |
| *≥12* | N = 56 | 54.7% [34.9%] | N = 42 | 57.1% [98.8%] |
| *p-value* | **0.04** | | 1.00 | |
|  |  | |  | |
| Completeness of cytoreduction |  | |  | |
| *CC-0* | N = 80 | 44.1% [33.6%] | N = 68 | 50.5% [76%] |
| *CC-1, 2 & 3* | N = 19 | 63.6% [33.7%] | N = 12 | 75.7% [94.8%] |
| *p-value* | 0.06 | | 1.00 | |
| *Categorical variables presented as frequency (percentage) and were analysed using Chi-squared test. Continuous variables presented as median, interquartile range [IQR] and range, and were analysed used Mann-Whitney test. Holm-Bonferroni test was used to adjust for multiple testing. Statistical significance is set at p<0.05. Significant p values are represented in bold.*  *.* | | | | |

| Patient characteristics and postoperative outcomes according to median preoperative functional capacity - with Holm-Bonferroni post-hoc correction | | | |
| --- | --- | --- | --- |
| 6MWT | | | |
| **Patient characteristics** | **6MWD < 510m**  **(n = 90)** | **6MWD ≥ 510m**  **(n = 107)** | **P value** |
|  |  |  |  |
| Age (years) | 61 [20] | 53 [18] | 0.1 |
|  |  |  |  |
| Sex |  |  | 0.27 |
| *Male* | 38 (42.2%) | 57 (53.3%) |  |
| *Female* | 52 (57.8%) | 50 (46.7%) |  |
|  |  |  |  |
| BMI | 28.3 [7.6] | 26.5 [6.8] | 0.4 |
|  |  |  |  |
| ASA score |  |  | **<0.001** |
| *1* | 2 (2.2%) | 6 (5.6%) |  |
| *2* | 23 (25.6%) | 58 (54.2%) |  |
| *3* | 65 (72.2%) | 43 (40.2%) |  |
|  |  |  |  |
| ECOG score |  |  | **<0.001** |
| *0* | 42 (46.7%) | 91 (85%) |  |
| *1* | 37 (41.1%) | 14 (13.1%) |  |
| *2* | 11 (12.2%) | 2 (1.9%) |  |
|  |  |  |  |
| Peritoneal Cancer Index  (PCI) | 15 [16.3] | 11 [15] | 0.18 |
|  |  |  |  |
| Completeness of cytoreduction |  |  | 0.27 |
| *CC-0* | 67 (74.4%) | 90 (84.1%) |  |
| *CC-1, 2 & 3* | 23 (25.6%) | 17 (15.9%) |  |
|  |  |  |  |
| Length of hospital stay  (days) | 21.5 [23] | 5 [8] | **<0.001** |
|  |  |  |  |
| Intensive care unit stay, (days) | 5 [3] | 5 [2.5] | **0.04** |
|  |  |  |  |
| Number of hospital readmissions |  |  | 0.12 |
| *0* | 45 (50%) | 69 (64.5%) |  |
| *≥1* | 45 (50%) | 38 (35.5%) |  |
|  |  |  |  |
| Discharge destination |  |  | **0.03** |
| *Home* | 74 (82.2%) | 102 (95.3%) |  |
| *Other (other hospital, rehabilitation, deceased in hospital)* | 16 (17.8%) | 5 (4.7%) |  |
|  |  |  |  |
| Postoperative complications |  |  | 0.6 |
| *Complications* | 62 (68.9%) | 77 (72%) |  |
| *No complications* | 28 (31.1%) | 30 (28%) |  |
|  |  |  |  |
| Clavien-Dindo |  |  | **0.04** |
| *I-II* | 33 (53.2%) | 58 (75.3%) |  |
| *III-V* | 29 (46.8%) | 19 (24.7%) |  |
|  |  |  |  |
| Comprehensive Complication Index (CCI) |  |  | 0.12 |
| *≤6* | 76 (84.4%) | 100 (93.5%) |  |
| *>6* | 14 (15.6% | 7 (6.5%) |  |
|  |  |  |  |
| 5STS | | | |
| **Patient characteristics** | **5STS < 9.3**  **(n = 96)** | **5STS ≥ 9.3s**  **(n = 96)** | **P values** |
|  |  |  |  |
| Age (years) | 52 [19.8] | 58.5 [16.8] | **0.04** |
|  |  |  |  |
| Sex |  |  | 0.6 |
| *Male* | 46 (47.9%) | 52 (54.2%) |  |
| *Female* | 50 (52.1%) | 44 (45.8%) |  |
|  |  |  |  |
| BMI | 26.9 [6.7] | 28.3 [7.7] | 0.6 |
|  |  |  |  |
| ASA score |  |  | 0.1 |
| *1* | 4 (4.2%) | 2 (2.1%) |  |
| *2* | 48 (50%) | 31 (32.3%) |  |
| *3* | 44 (45.8%) | 63 (65.6%) |  |
|  |  |  |  |
| ECOG score |  |  | **0.04** |
| *0* | 75 (78.1%) | 54 (56.3%) |  |
| *1* | 17 (17.7%) | 33 (34.4%) |  |
| *2* | 4 (4.2%) | 9 (9.4%) |  |
|  |  |  |  |
| Peritoneal Cancer Index  (PCI) | 11 [12.8] | 15 [20] | 0.1 |
|  |  |  |  |
| Completeness of cytoreduction |  |  | 0.1 |
| *CC-0* | 83 (86.5%) | 70 (72.9%) |  |
| *CC-1, 2 & 3* | 13 (13.5%) | 26 (27.1%) |  |
|  |  |  |  |
| Length of hospital stay  (days) | 16 [10] | 20 [14] | 0.2 |
|  |  |  |  |
| Intensive care unit stay (days) | 5 [2] | 5 [2.75] | 0.5 |
|  |  |  |  |
| Number of hospital readmissions |  |  | **0.01** |
| *0* | 67 (69.8%) | 46 (47.9%) |  |
| *≥1* | 29 (30.2%) | 50 (52.1%) |  |
|  |  |  |  |
| Discharge destination |  |  | 0.1 |
| *Home* | 91 (94.8%) | 80 (83.3%) |  |
| *Other (other hospital, rehabilitation, deceased in hospital)* | 5 (5.2%) | 16 (16.7%) |  |
|  |  |  |  |
|  |  |  |  |
| Postoperative complications |  |  | 0.5 |
| *Complications* | 72 (75%) | 65 (67.7%) |  |
| *No complications* | 24 (25%) | 31 (32.3%) |  |
|  |  |  |  |
| *Clavien-Dindo* |  |  | 0.5 |
| *I-II* | 51 (70.8%) | 39 (60%) |  |
| *III-V* | 21 (29.2%) | 26 (40%) |  |
| Comprehensive Complications Index (CCI) |  |  | 0.1 |
| *≤6* | 91 (94.8%) | 81 (84.4%) |  |
| *>6* | 5 (5.2%) | 15 (15.6%) |  |
| *Categorical variables presented as frequency (percentage) and were analysed using Chi-squared test. Continuous variables presented as median, interquartile range [IQR] and range, and were analysed used Mann-Whitney test. Holm-Bonferroni test was used to adjust for multiple testing. Statistical significance is set at p<0.05. Significant p values are represented in bold.* | | | |

| 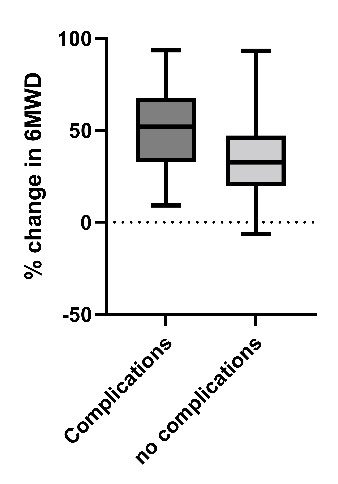  **A**  **B**  **F** | 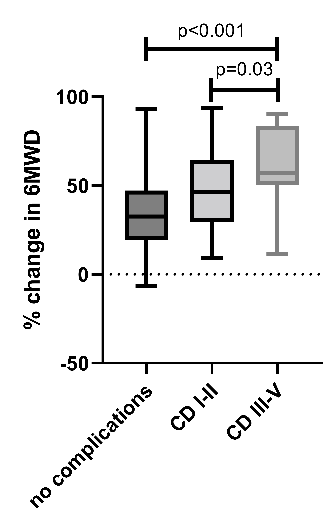  **E**  **G** | 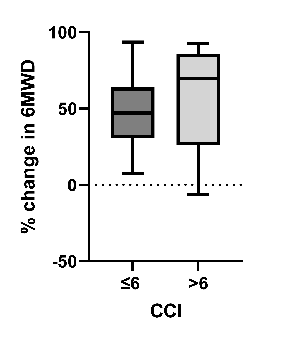  **D**  **C**  **H** | 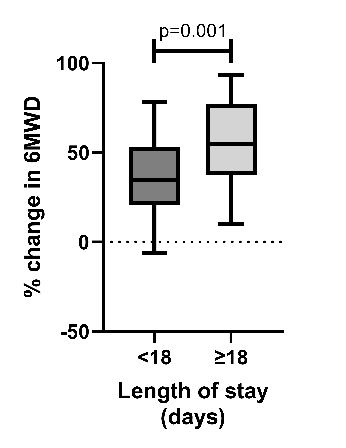 |
| --- | --- | --- | --- |
| 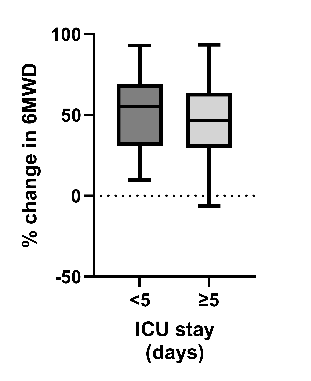  **I**  **J** | 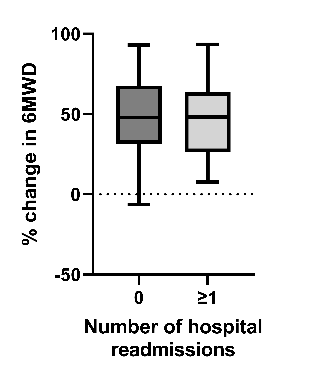  **K** | 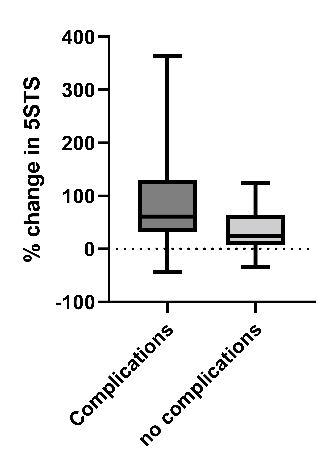  **L** | 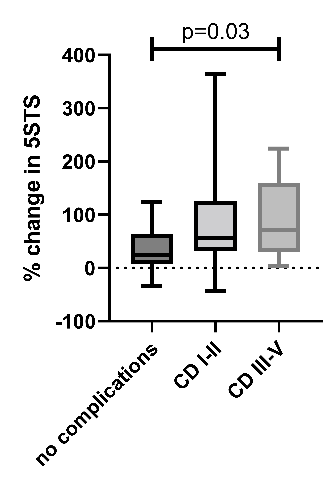 |
| 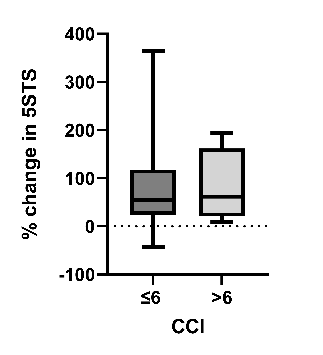 | 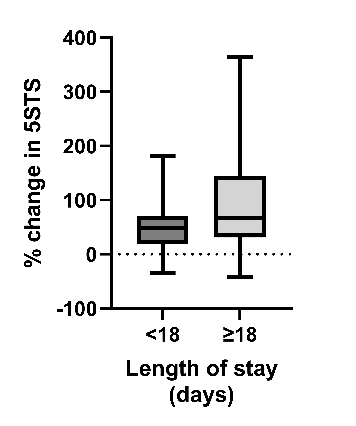 | 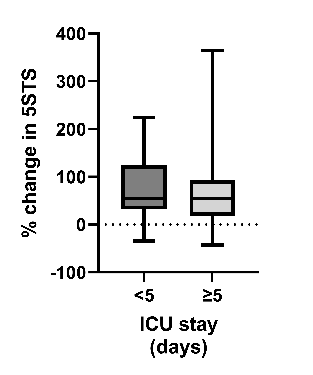 | 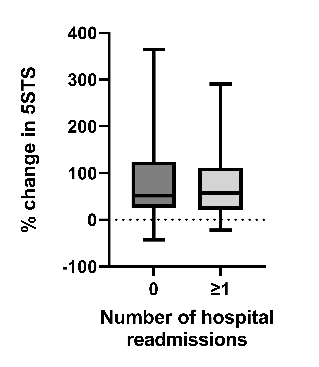 |
| Association between percentage change in physical function, and postoperative outcomes, analysed with Holm-Bonferroni post-hoc correction. Percentage change in function was calculated for six-minute walk distance (6MWD) with the following formula: [(preoperative 6MWD – postperative 6MWD)/preoperative 6MWD]*100. Percentage change in function was calculated for five-times sit to stand test (5STS) with the following formula: [(postperative 5STS – preoperative 5STS)/preoperative 5STS]*100. Graphs A to F show percentage change in 6MWD, and graphs D to F represent percentage change in 5STS, analysed based on complications (occurrence, Clavien Dindo (CD) and Comprehensive Complication Index (CCI)), length of hospital stay (LOS), ICU stay and number of hospital readmissions. Continuous variables were dichotomised based on the median value. Data is presented as median and interquartile range (error bars show minimum and maximum). Statistical significance is determined using the Mann-Whitney test with Holm-Bonferonni post-hoc correction or Kruskal Wallis with Dunn’s multiple comparisons test. Significance is set at p<0.05. | | | |
