# Supplementary material for: Functional Outcomes Following Cytoreductive Surgery and Hyperthermic Intraperitoneal Chemotherapy: A Prospective Cohort Study
Source: Ann Surg Oncol. 2022 Oct 28;30(1):447–58. doi: 10.1245/s10434-022-12691-x (PMC9726807; doi:10.1245/s10434-022-12691-x)
Supplement: Supplementary file 3 — Supplementary file3 (DOCX 21 kb) [file 10434_2022_12691_MOESM3_ESM.docx]

| **Supplementary Table 1:** Percentage change in physical function following cytoreductive surgery, based on patient characteristics, oncological and surgical factors | | | | | | |
| --- | --- | --- | --- | --- | --- | --- |
| **Preoperative patient characteristics** | **% Change in 6MWD**  **(n = 99)** | | | **% Change in 5STS**  **(n = 80)** | | |
| Age (years) |  | | |  | | |
| *<56* | N = 50 | 47.7% [34.6%]] | | N = 44 | 48% [110.7%] | |
| *≥56* | N = 49 | 48.1% [36.5%] | | N = 36 | 57.1% [63.6%] | |
| *p-values* | 0.74 | | | 0.78 | | |
|  |  | | |  | | |
| Sex |  | | |  | | |
| *Male* | N = 44 | 36.8% [39.8%] | | N = 34 | 49.9% [55.7%] | |
| *Female* | N = 55 | 53% [30.6%] | | N = 46 | 59.3% [109.4%] | |
| *p-values* | 0.07 | | | 0.42 | | |
|  |  | | |  | | |
| BMI |  | | |  | | |
| *<27.9* | N = 52 | 47.7% [32.4%] | | N = 43 | 49.7% [48.8%] | |
| *≥27.9* | N = 47 | 48.6% [37.7%] | | N = 37 | 64.8% [108%] | |
| *p-values* | 0.88 | | | 0.39 | | |
|  |  | | |  | | |
| ASA score |  | | |  | | |
| *1* | N = 2 | 53.1% [12.7%] | | N = 2 | 67.8% [7.6%] | |
| *2* | N = 41 | 47.2% [38.1%] | | N = 36 | 54.5% [92.9%] | |
| *3* | N = 56 | 48.3% [36%] | | N = 42 | 49.8% [73%] | |
| *p-value* | 0.65 | | | 0.30 | | |
|  |  | | |  | | |
| ECOG score |  | | |  | | |
| *0* | N = 67 | 45.9% [31.2%] | | N = 57 | 51.4% [67.9%] | |
| *1* | N = 29 | 50% [36.4%] | | N = 22 | 66.7% [103.2%] | |
| *2* | N = 3 | 82.2% [32%] | | N = 1 | 4.2% [0%] | |
| *p-value* | 0.14 | | | 0.28 | | |
|  |  | | |  | | |
| Peritoneal cancer index  (PCI) |  | | |  | | |
| *<12* | N = 43 | 36.8% [33.9%] | | N = 38 | 48.2% [67%] | |
| *≥12* | N = 56 | 54.7% [34.9%] | | N = 42 | 57.1% [98.8%] | |
| *p-value* | **0.004** | | | 0.45 | | |
|  |  | | |  | | |
| Completeness of cytoreduction |  | | |  | | |
| *CC-0* | N = 80 | 44.1% [33.6%] | | N = 68 | 50.5% [76%] | |
| *CC-1, 2 & 3* | N = 19 | 63.6% [33.7%] | | N = 12 | 75.7% [94.8%] | |
| *p-value* | **0.007** | | | 0.16 | | |
|  |  | | |  | | |
| HIPEC |  | | |  | | |
| *Mitomycin-C* | N = 67 | | 46.4% [34.9%] | N = 56 | | 55.6% [98.9%] |
| *Platinum (Cisplatin or Oxaliplatin)* | N = 22 | | 45% [30.6%] | N = 15 | | 45.3% [53.9%] |
| *p-value* | 0.44 | | | 0.12 | | |
| *Categorical variables presented as frequency (number of patients). Continuous variables presented as median and interquartile range [IQR]. Statistical analysis performed using the Mann-Whitney test or Kruskal-Wallis test. Statistical significance is set at p<0.05. Significant p values are represented in bold.* | | | | | | |
