# Supplementary material for: Functional Outcomes Following Cytoreductive Surgery and Hyperthermic Intraperitoneal Chemotherapy: A Prospective Cohort Study
Source: Ann Surg Oncol. 2022 Oct 28;30(1):447–58. doi: 10.1245/s10434-022-12691-x (PMC9726807; doi:10.1245/s10434-022-12691-x)
Supplement: Supplementary file 4 — Supplementary file4 (DOCX 28 kb) [file 10434_2022_12691_MOESM4_ESM.docx]

| **Supplementary Table 2:** Patient characteristics and postoperative outcomes according to median preoperative functional capacity | | | |
| --- | --- | --- | --- |
| 6MWT | | | |
| **Patient characteristics** | **6MWD < 510m**  **(n = 90)** | **6MWD ≥ 510m**  **(n = 107)** | **P value** |
|  |  |  |  |
| Age (years) | 61 [20] | 53 [18] | **0.02** |
|  |  |  |  |
| Sex |  |  | 0.12 |
| *Male* | 38 (42.2%) | 57 (53.3%) |  |
| *Female* | 52 (57.8%) | 50 (46.7%) |  |
|  |  |  |  |
| BMI | 28.3 [7.6] | 26.5 [6.8] | 0.4 |
|  |  |  |  |
| ASA score |  |  | **<0.001** |
| *1* | 2 (2.2%) | 6 (5.6%) |  |
| *2* | 23 (25.6%) | 58 (54.2%) |  |
| *3* | 65 (72.2%) | 43 (40.2%) |  |
|  |  |  |  |
| ECOG score |  |  | **<0.001** |
| *0* | 42 (46.7%) | 91 (85%) |  |
| *1* | 37 (41.1%) | 14 (13.1%) |  |
| *2* | 11 (12.2%) | 2 (1.9%) |  |
|  |  |  |  |
| Peritoneal Cancer Index  (PCI) | 15 [16.3] | 11 [15] | **0.046** |
|  |  |  |  |
| Completeness of cytoreduction |  |  | 0.09 |
| *CC-0* | 67 (74.4%) | 90 (84.1%) |  |
| *CC-1, 2 & 3* | 23 (25.6%) | 17 (15.9%) |  |
|  |  |  |  |
| Length of hospital stay  (days) | 21.5 [23] | 5 [8] | **<0.001** |
|  |  |  |  |
| Intensive care unit stay, (days) | 5 [3] | 5 [2.5] | **0.008** |
|  |  |  |  |
| Number of hospital readmissions |  |  | **0.04** |
| *0* | 45 (50%) | 69 (64.5%) |  |
| *≥1* | 45 (50%) | 38 (35.5%) |  |
|  |  |  |  |
| Discharge destination |  |  | **0.005** |
| *Home* | 74 (82.2%) | 102 (95.3%) |  |
| *Other (other hospital, rehabilitation, deceased in hospital)* | 16 (17.8%) | 5 (4.7%) |  |
|  |  |  |  |
| Postoperative complications |  |  | 0.6 |
| *Complications* | 62 (68.9%) | 77 (72%) |  |
| *No complications* | 28 (31.1%) | 30 (28%) |  |
|  |  |  |  |
| Clavien-Dindo |  |  | **0.007** |
| *I-II* | 33 (53.2%) | 58 (75.3%) |  |
| *III-V* | 29 (46.8%) | 19 (24.7%) |  |
|  |  |  |  |
| Comprehensive Complication Index (CCI) |  |  | **0.04** |
| *≤6* | 76 (84.4%) | 100 (93.5%) |  |
| *>6* | 14 (15.6% | 7 (6.5%) |  |
|  |  |  |  |
| 5STS | | | |
| **Patient characteristics** | **5STS < 9.3**  **(n = 96)** | **5STS ≥ 9.3s**  **(n = 96)** | **P values** |
|  |  |  |  |
| Age (years) | 52 [19.8] | 58.5 [16.8] | **0.006** |
|  |  |  |  |
| Sex |  |  | 0.4 |
| *Male* | 46 (47.9%) | 52 (54.2%) |  |
| *Female* | 50 (52.1%) | 44 (45.8%) |  |
|  |  |  |  |
| BMI | 26.9 [6.7] | 28.3 [7.7] | 0.3 |
|  |  |  |  |
| ASA score |  |  | **0.02** |
| *1* | 4 (4.2%) | 2 (2.1%) |  |
| *2* | 48 (50%) | 31 (32.3%) |  |
| *3* | 44 (45.8%) | 63 (65.6%) |  |
|  |  |  |  |
| ECOG score |  |  | **0.005** |
| *0* | 75 (78.1%) | 54 (56.3%) |  |
| *1* | 17 (17.7%) | 33 (34.4%) |  |
| *2* | 4 (4.2%) | 9 (9.4%) |  |
|  |  |  |  |
| Peritoneal Cancer Index  (PCI) | 11 [12.8] | 15 [20] | **0.049** |
|  |  |  |  |
| Completeness of cytoreduction |  |  | **0.02** |
| *CC-0* | 83 (86.5%) | 70 (72.9%) |  |
| *CC-1, 2 & 3* | 13 (13.5%) | 26 (27.1%) |  |
|  |  |  |  |
| Length of hospital stay  (days) | 16 [10] | 20 [14] | **0.04** |
|  |  |  |  |
| Intensive care unit stay (days) | 5 [2] | 5 [2.75] | 0.16 |
|  |  |  |  |
| Number of hospital readmissions |  |  | **0.002** |
| *0* | 67 (69.8%) | 46 (47.9%) |  |
| *≥1* | 29 (30.2%) | 50 (52.1%) |  |
|  |  |  |  |
| Discharge destination |  |  | **0.02** |
| *Home* | 91 (94.8%) | 80 (83.3%) |  |
| *Other (other hospital, rehabilitation, deceased in hospital)* | 5 (5.2%) | 16 (16.7%) |  |
|  |  |  |  |
| Postoperative complications |  |  | 0.2 |
| *Complications* | 72 (75%) | 65 (67.7%) |  |
| *No complications* | 24 (25%) | 31 (32.3%) |  |
|  |  |  |  |
| *Clavien-Dindo* |  |  | 0.18 |
| *I-II* | 51 (70.8%) | 39 (60%) |  |
| *III-V* | 21 (29.2%) | 26 (40%) |  |
| Comprehensive Complications Index (CCI) |  |  | **0.02** |
| *≤6* | 91 (94.8%) | 81 (84.4%) |  |
| *>6* | 5 (5.2%) | 15 (15.6%) |  |
| *Categorical variables presented as frequency (percentage). Continuous variables presented as median and interquartile range [IQR]. Statistical significance is set at p<0.05. Significant p values are represented in bold.* | | | |
